# Machine Learning and Wearable Technology: Monitoring Changes in Biomedical Signal Patterns during Pre-Migraine Nights

**DOI:** 10.3390/healthcare12171701

**Published:** 2024-08-26

**Authors:** Viroslava Kapustynska, Vytautas Abromavičius, Artūras Serackis, Šarūnas Paulikas, Kristina Ryliškienė, Saulius Andruškevičius

**Affiliations:** 1 Faculty of Electronics, Vilnius Gediminas Technical University, Plytinės st. 25, 10105 Vilnius, Lithuaniaarturas.serackis@vilniustech.lt (A.S.); sarunas.paulikas@vilniustech.lt (Š.P.); 2 Center of Neurology, Vilnius University, Santariškių st. 2, 08406 Vilnius, Lithuania; kristina.ryliskiene@santa.lt (K.R.); saulius.andruskevicius@santa.lt (S.A.)

**Keywords:** migraine prediction, machine learning, wearable biosensors, signal feature extraction, feature ranking, sleep analysis, nocturnal monitoring

## Abstract

Migraine is one of the most common neurological disorders, characterized by moderate-to-severe headache episodes. Autonomic nervous system (ANS) alterations can occur at phases of migraine attack. This study investigates patterns of ANS changes during the pre-ictal night of migraine, utilizing wearable biosensor technology in ten individuals. Various physiological, activity-based, and signal processing metrics were examined to train predictive models and understand the relationship between specific features and migraine occurrences. Data were filtered based on specified criteria for nocturnal sleep, and analysis frames ranging from 5 to 120 min were used to improve the diversity of the training sample and investigate the impact of analysis frame duration on feature significance and migraine prediction. Several models, including XGBoost (Extreme Gradient Boosting), HistGradientBoosting (Histogram-Based Gradient Boosting), Random Forest, SVM, and KNN, were trained on unbalanced data and using cost-sensitive learning with a 5:1 ratio. To evaluate the changes in features during pre-migraine nights and nights before migraine-free days, an analysis of variance (ANOVA) was performed. The results showed that the features of electrodermal activity, skin temperature, and accelerometer exhibited the highest F-statistic values and the most significant *p*-values in the 5 and 10 min frames, which makes them particularly useful for the early detection of migraines. The generalized prediction model using XGBoost and a 5 min analysis frame achieved 0.806 for accuracy, 0.638 for precision, 0.595 for recall, and 0.607 for F1-score. Despite identifying distinguishing features between pre-migraine and migraine-free nights, the performance of the current model suggests the need for further improvements for clinical application.

## 1. Introduction

Migraine is a complex neurovascular disorder that affects more than a billion people worldwide, making it one of the most prevalent neurological conditions globally [1]. The impact of migraine extends far beyond the physical pain it causes, negatively influencing several critical aspects of an individual’s life such as marriage, parenting, and family relationships, as well as career prospects, financial stability, and general health [2]. The unpredictable nature of migraine attacks is particularly distressing for sufferers, as highlighted in numerous studies [3,4,5,6]. Consequently, individuals suffering from migraines are three times more likely to develop generalized anxiety disorders compared to the general population.

The desire for better management and predictability of migraine attacks has been clearly expressed by those affected. A survey of 565 people concluded that 88.8% would like a device to predict migraine attacks, most preferring a wrist-worn option [7]. Almost all participants in another study [8] agreed that they would want information on when a migraine might occur. With the advent of personal health tools such as apps, smartphones, and wearable devices, it is now possible to capture objective and prospective data on a large scale. This development facilitates the collection of massive datasets that can be used to develop prediction models with less effort and cost, enabling studies at both the individual and population levels. The biomedical signals measured by wearable devices can provide valuable information on physiological changes, such as variations in skin temperature (SkinTEMP), pulse, and sleep quality, offering insights into changes that occur before the onset of migraines.

In this study, we used the newer Empatica Embrace Plus device [9], which offers enhanced features over the commonly used Empatica E4 [10,11,12,13]. Our focus is on understanding pre-migraine symptoms, which could lead to earlier interventions and better patient outcomes [14]. However, the reliability of triggers in predicting an attack varies between and within individuals, adding complexity to the development of prediction models [15].

According to the third edition of the International Classification of Headache Disorders (ICHD-3) [16], some patients experience various prodromal symptoms that can start up to two days before the onset of headache. In the PRODROME trial [17], scientists analyzed prodromal symptoms in 920 participants who stated that they could identify migraine attacks with prodromal symptoms reliably followed by headache and showed that treatment during the prodrome prevents the onset of moderate or severe headaches and reduces functional disability. Clarifying our understanding of pre-headache symptoms could improve our ability to study the underlying mechanisms of attack onset and may lead to earlier treatment during the prodrome, with the intention of shortening the prodrome, preventing headaches, and improving function. Given the complex nature of migraine attacks, more studies are required at the individual and population levels [14].

Studies have shown that changes in biomedical signals regulated by the autonomic nervous system (ANS) are significant in chronic diseases, including migraine [18]. Various signal processing techniques and machine learning models have been explored to predict migraines [19,20,21,22].

AI models have shown good results in the detection and classification of headache disorders, including migraine, using deep learning and ML techniques [23].

Among the relatively small number of studies focused on migraine prediction, we selected those that used monitoring through electronic journals [17,24], wearable sensors [8,10,11,12,13,25], finger-held peripheral capillary oxygen saturation devices (SpO2), and ECG patches [18]. These studies are notable for their data collection methods that involve migraine participants and their significant contributions to the development of migraine prediction models [12,18,25,26,27].

Researchers who have conducted studies on migraine prediction have identified specific biomedical signals that are considered particularly informative in the prediction of migraine episodes. For example, in their work in [18], the authors highlighted the importance of a multivariate analysis involving the HR, EDA, SkinTEMP, and SpO2 signals for the prediction of migraine.

To collect their data, the researchers used an SpO2 finger-sized device and affixed ECG patches. During their investigation, the authors of [18] observed that there was a lack of sufficiently useful models for migraine prediction, except in cases involving the combination of SkinTEMP, HR, and SpO2. In this combination, they found that four different models performed reasonably well in predicting migraine attacks in two participants with migraine. However, the prediction accuracy using the combination of SkinTEMP, EDA, HR, and SpO2 exhibited a relatively high false positive rate (FPR) in their investigation.

In the study in [25], the authors included eight participants with episodic migraine and recorded real-time hemodynamic signals, including TEMP, HR, and EDA, obtained from a wrist-wearable device for 24 h. Personalized prediction models were generated using artificial recurrent neural networks, such as long short-term memory (LSTM), to compute on a one-minute basis if pain would appear in the next 120 min. Data were balanced in pain–no-pain time periods to train the models. The algorithm was able to predict migraine attacks with a sensitivity of 95% throughout the sample.

The study in [12] used the Empatica E4 wristband to collect data from seven volunteers with different types of migraine. What distinguishes this research from previous studies is its exclusive focus on data collected during sleep periods. The authors chose this approach based on findings from a prior study [13], which highlighted the impact of physical activity on heart rate (HR).

The fundamental hypothesis of the study in [12] posits that focusing on the signals acquired during sleep allows a rough estimate of the probability that a migraine attack occurs the following day. To test this hypothesis, sleep data were classified into two classes: (1) nights preceding a day without a migraine, and (2) nights preceding days with a migraine. Consequently, class (2) contained information related to the prodromal stage of a migraine attack. The features used in the analysis included accelerometers (Accs), blood volume pulse (BVP), TEMP, EDA, RR, and pulse rate variability (PRV).

In a study, quadratic discriminant analysis (QDA) and linear discriminant analysis (LDA) were compared, and the authors found that QDA produces better results than LDA (84.1% vs. 70.2% in personal recognition models). The authors of [12] also tested user-independent recognition models, but found that they could not detect migraine attacks, the balanced recognition accuracy was below 50%. To gain a better understanding of how well a user-independent model can detect migraine attacks in advance, data should be collected from much larger user groups, or the study should focus only on one type of migraine attack [12].

The body of research that confirms changes in biomedical signals during migraine episodes, together with the presence of observable prodromal symptoms in some individuals before the onset of a migraine, coupled with intriguing findings from other studies in the field of migraine prediction, strongly suggests the potential for abnormalities in biomedical signals during the prodromal phase. This hypothesis has driven our investigation, where we leveraged the expertise of colleagues who have conducted similar research on migraine using modern analytical tools and technologies.

The main contributions of this paper are outlined as follows:

1. A comprehensive investigation into the application of wearable technology in conjunction with machine learning models to predict migraine attacks by analyzing physiological signals collected during nocturnal sleep.
2. A detailed examination of the effectiveness of various analysis frames in predicting migraine attacks, highlighting the most suitable time frames for accurate predictions.
3. The identification of key physiological features with the highest predictive power for detecting abnormalities that precede migraine onset, providing valuable insights into the early detection of migraines.
4. Establishing a foundation for future research by emphasizing the importance of traditional statistical metrics in feature extraction and suggesting the integration of physiological data collected over the investigated periods to improve prediction models.

## 2. Methods

**Figure 1:**
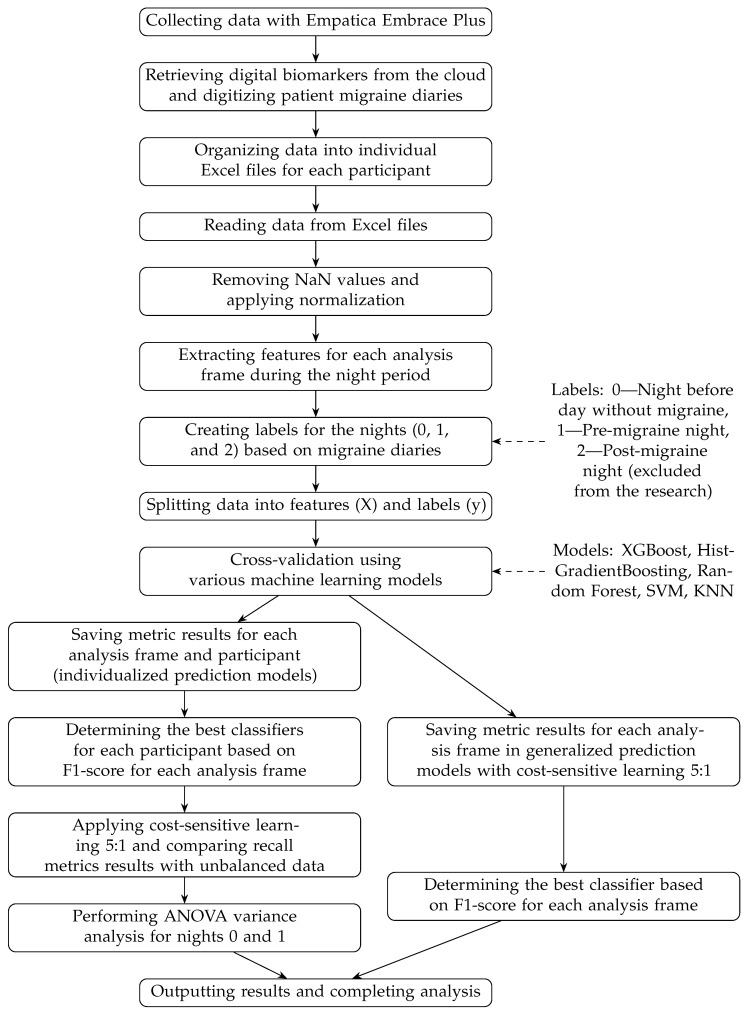
Workflow diagram of the research process.

Inspiration for this investigation was drawn from the study in [12], leading to the decision to conduct a similar investigation using biomedical signals from nocturnal sleep periods. Consequently, a labeling approach was adopted for nights based on participants’ migraine diaries, where nights preceding a day without migraines were designated as (0) and pre-migraine nights as (1); nights after a day with migraine were labeled as (2) and excluded from the investigation due to possible abnormalities. These labels were used as output in the developed ML model. The input comprised 78 features, extracted from six signals measured by the Empatica Embrace Plus wristband. A general workflow diagram of the research process is shown in Figure 1.

An important aspect of this research involved the implementation of precise criteria to define nocturnal sleep data and fit them into frames of 5, 10, 30, 60, 90, and 120 min for analysis. We used this method to expand the dataset and explore how the duration of the analysis frame impacted the precision of migraine prediction.

In the study, specific criteria were established to define nocturnal sleep data. These criteria were based on the time interval between 7 p.m. and 8 a.m., and values of the sleep detection stage (SDS) measured by Empatica Embrace Plus, which help identify different phases of sleep and limit night movements to no more than 20 steps, and require the SDS value to be exactly 101, which strictly indicates sleeping.

After identifying the nocturnal sleep data, they were subdivided into analysis frames, upon which feature extraction was subsequently performed. This approach was essential for the operation of the developed framework and the sleep-based ML model.

### 2.1. Empatica Embrace Plus Wristband

The Empatica Embrace Plus [9] enables extended data collection of up to 14 days due to its long battery life and increased NOR flash memory, with rapid 90 min charging for longer monitoring cycles, essential to our study.

Featuring multiple sensors, including a 3D accelerometer, gyroscope, PPG sensor (up to 64 Hz), TEMP sensor (1 Hz), and EDA sensor (up to 4 Hz), the device provides access to raw data, digital biomarkers, and reports via the Empatica Health Monitoring Platform.

Embrace Plus offers four configurations: Pulse Rate Pro, SpO2 Pro, Actigraphy Pro, and Actigraphy Optimized. Its algorithms derive digital biomarkers from raw sensor data, such as RMSSD for PRV, respiratory rate from PPG and Acc data, and continuous monitoring of SpO2.

Additional algorithms include those for SDS, activity count (ACT), activity classification, body position, and MET, using the 3-axis accelerometer; along with gait speed, skin conductance level, and continuous SkinTEMP estimation from EDA sensors. These biomarkers capture vital physiological data, although SpO2 is not included in our professional plan.

In reviewing Empatica Embrace Plus, we focus on the precision of its HR algorithm during the daytime that was questioned in the study [13,25]. The developers [28] reported that, under no motion conditions, the error of the PR algorithm is less than 3 bpm, and under typical daily activities, less than 5 bpm, both within clinically acceptable ranges. Although our study uses primarily nocturnal sleep data, these findings support the reliability of using PR data from daytime activity for further research.

In this study, digital biomarkers obtained from the device were utilized, providing insight into various physiological parameters. The signals used, their physical significance, and units of measurement are described in the following.

- Pulse rate (PR): Continuous monitoring of the participant’s heart rate, measured in beats per minute (bpm).
- Pulse rate variability—RMSSD: Intermittent monitoring of pulse rate variability, expressed as the root-mean-square of successive differences between consecutive systolic peaks, measured in milliseconds (ms).
- Respiratory rate (RR): Intermittent monitoring of the participant’s breathing rate, measured in breaths per minute (brpm).
- Sleep detection: Automatic detection of sleep periods based on physical activity data.
- Activity count: Continuous estimation of movement intensity, measured in arbitrary units (a.U.).
- Accelerometer magnitude standard deviation: Average standard deviation of Acc data magnitude, measured in g (gravitational force).
- Step count: Continuous monitoring of step count, measured in steps.
- Metabolic equivalent of task (MET): Continuous estimation of energy consumption during physical activity, measured in MET.
- Electrodermal activity (EDA): Continuous monitoring of the participant’s skin conductance level, measured in microsiemens (μ siemens).
- Wearing detection: Detection of device wearing status, expressed as the proportion of time the device is worn versus not worn, measured in percentage (%).

### 2.2. Participants

In this study, ten migraine sufferers were enrolled according to specific inclusion criteria to assess the applicability of wearable biosensor technology to predict migraine attacks by monitoring changes in ANS during the prodrome phase. Participants were instructed to wear the device on the wrist of their non-dominant hand and continue using it until the occurrence of at least three migraine episodes was recorded. The demographic information and the number of monitoring days for each participant are shown in Table 1.

To minimize external impacts on changes in the autonomic nervous system for the most precise analysis possible, and to avoid bioethical concerns, subjects were included according to the following inclusion and exclusion criteria.

#### 2.2.1. Inclusion Criteria

- Age ≥ 18 years;
- Diagnosis of episodic migraine with/without aura, meeting ICHD-3 (3rd International Classification of Headache Disorders) criteria;
- Frequency of at least 4 migraine attacks per month;
- Ability to write, read, and understand the Lithuanian language;
- Ability to understand and perform all procedures defined in the study protocol.

#### 2.2.2. Exclusion Criteria

- Pregnant and lactating women;
- Diagnosis of chronic and/or hemiplegic migraine;
- Diagnosis of other headache syndrome, except for episodic tension-type headache whose frequency does not exceed 4 days per month;
- Use of preventive migraine treatment;
- Use of drugs with an effect on the ANS: antidepressants, cholinergic and anticholinergic drugs, antipsychotics, antihistamines, antihypertensives, antiepileptic drugs, opioids, benzodiazepines, antiparkinsonian drugs, antispasmodics, antiemetics, muscle relaxants;
- Other diagnosis of chronic pain.

### 2.3. Data Pre-Processing

During the pre-processing stage of the study data, the objective was to develop individual datasets for migraine prediction models for each participant. The digital biomarkers, systematically organized into daily folders containing 14 files corresponding to different signals in the cloud, were merged into a single comprehensive file per participant.

Table 2 shows that PRV and RR signals were excluded from the research due to their high proportion of missing values. This pattern of a high percentage of missing data in the PRV and RR signals was consistently observed across all participants’ data.

The data pre-processing involved removing missing values to ensure that the data were reliable and complete. Data were normalized according to the formula

(1) $$\mathrm{Normalized}\;\mathrm{Signal}=\frac{\mathrm{Signal}-\min(\mathrm{Signal})}{\max(\mathrm{Signal})-\min(\mathrm{Signal})}.$$

**Figure 2:**
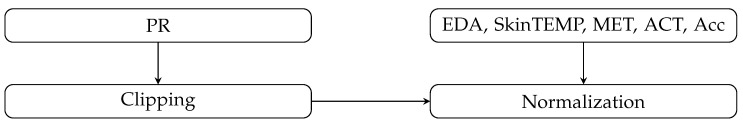
Data pre-processing workflow.

To eliminate non-informative values in the PR signal resulting from issues such as poor device attachment, the values were clipped to predefined thresholds of 25 to 115 bpm (Figure 2).

The features were extracted using statistical measures described in the section Feature Extraction. As mentioned above and in Figure 1, each night was labeled 0, 1, or 2. Several classifiers were used: Random Forest (100 estimators, Gini criterion), HistGradientBoosting (learning rate 0.1, max depth 3, min samples leaf 20), XGBoost (learning rate 0.1, 100 estimators, max depth 3, mlogloss metric), support vector machine (SVM) algorithm (C = 1.0, RBF kernel, scale gamma), and k-nearest neighbors algorithm (KNN) (5 neighbors, uniform weights, Minkowski metric). The SVM algorithm classifies the extracted features by identifying the optimal hyperplane that maximizes the margin between migraine and non-migraine classes in our provided feature space, as used for similar classification tasks [29]. In contrast, XGBoost, a gradient boosting algorithm, iteratively updates decision trees, optimizes accuracy by reducing prediction errors, and effectively captures complex patterns within the data [30].

Stratified five-fold cross-validation ensured robust evaluation, calculating accuracy, precision, recall, and F1-score. This approach involves dividing the dataset into five equal parts, with each part serving as a test set once and as part of the training set four times. This cycling ensures that each data segment is used for both training and testing [31].

Given the dataset imbalance, random under-sampling was applied as a part of the research process to achieve a 5:1 ratio using random under-sampling, and recall metrics that were received were compared while using unbalanced data.

An ANOVA variance analysis, which is widely used in medical studies [32,33,34], was performed to compare the characteristics between nights labeled 0 and 1. The classifiers were trained and evaluated based on F1-scores for different analysis frames, with the best classifiers summarized in the results tables.

## 3. Feature Extraction

The feature extraction phase was dedicated to data preparation for subsequent analysis and ML applications, which involves extraction of features from biomedical signals, including EDA, PR, Acc, MET, SkinTEMP, and ACT.

As mentioned above, nocturnal sleep was divided into analysis frames to indicate the duration of the analysis frame that was most suitable for the prediction of migraine attacks. For each of these analysis intervals, a set of features was extracted, including the following.

- Mean: The average level of the signal.
- Median: The central tendency of the data.
- Std: The degree of data variability relative to the mean.
- Max: The highest recorded value.
- Min: The lowest recorded value.
- Clearance factor: Reflects the maximum clearance in the data.
- Crest factor: Provides information on signal peaks.
- Impulse factor: Indicates abrupt changes in the signal.
- Kurtosis: Measures the distribution’s sharpness or flatness.
- Peak value: The highest peak in the data.
- RMS (root mean square): Magnitude of the signal.
- Shape factor: Provides insights into the shape of the signal.
- Skewness: Measures the symmetry or asymmetry of the data distribution.

These features were calculated using formulas from [35]. Time-domain features such as mean, median, Std, min, and max are used in similar research using wearables, for example, stress detection using Empatica E4 [36] and migraine forecast [12,37]. Other time-domain features, such as clearance factor, crest factor, impulse factor, peak value, kurtosis, RMS, shape factor, and skewness are successfully implemented in other domains [38,39]. The latter features are considered more explicable, but are not commonly used in data obtained from wearables [40,41]. This approach facilitated an exploration of the relationship between these features and migraine occurrences, providing insight into the predictive patterns of migraines.

In the learning process, the input is made up of 78 features, including 13 previously specified features for each of the six unique signals. These features are important ground for the development of classifiers, which involves training and evaluating a variety of ML models. The result of this method is a night-labeling system.

## 4. Results

### 4.1. Feature Ranking and Analysis

In the analysis performed, a variety of physiological, activity, and signal processing measurements were evaluated to determine their relative importance in predicting migraines. ANOVA feature ranking was used to evaluate the impact of the features on the prediction results. The recurrence of the signal source for the subset of features is shown in Table 3. The recurrence of features, independent of the signal source, is shown in Table 4.

Table 3 presents the recurrence results for each signal as a source within the subset of features ranked. From the initial set of 78 features, the 35 most significant were selected using analysis of variance (ANOVA), allowing us to identify the characteristics that have the most substantial impact on the predictive model. The PR and SkinTEMP signals showed the highest frequency of recurrence, highlighting their significant contribution to the overall set of features. In contrast, signals such as ACT and Acc appeared less frequently, indicating their relatively lower influence on the predictive model.

The analysis of the rank of characteristics, as illustrated in Table 4, provides information on how to predict migraine. Features such as median and min were frequently selected for a feature subset, independent of the signal source. In contrast, features such as skewness and kurtosis were less important, indicating that the shape of the data distribution might play a minor role in the prediction of migraine.

### 4.2. Classifier Performance Analysis

To evaluate the migraine prediction model, key metrics including accuracy, F1-score, precision, and recall were used. The analysis focused on different analysis frames, with the objective of identifying the best-performing classifier for each interval based on these metrics.

To define the most suitable classifier for each length of analysis frame, an experiment was conducted training XGBoost, HistGradientBoosting, Random Forest, SVM, and KNN models. Following this, the best ML model was chosen from the list above for each analysis frame based on the highest F1-score.

Random Forest shows strong and consistent performance across multiple participants and analysis frames. XGBoost also performs well, particularly in shorter analysis frames. HistGradientBoosting is another strong contender, particularly in medium to longer analysis frames.

Performance metrics vary significantly between participants. This could be due to individual differences in physiological signals or different levels of data quality and quantity. For some participants, a specific classifier consistently performs better, while for others, the best classifier changes with the duration of the analysis frame.

Shorter analysis frames tend to produce higher F1-scores and recall for most participants, as shown in Table 5 and Table 6. This is likely because shorter frames can capture more granular changes in physiological signals. The medium analysis frames show balanced performance, as demonstrated in Table 7 and Table 8, with some drop in F1-score and recall compared to shorter frames, but they are still useful. Longer analysis frames generally result in lower performance metrics; see Table 9 and Table 10. The ability to detect differences between migraine and non-migraine days diminishes with longer frames.

The 5 and 10 min frames are generally the best for achieving high F1-scores and recall. These shorter frames capture more detailed variations in the data, which is important for detecting the onset of migraines.

### 4.3. Rationale for Implementing Cost-Sensitive Learning in Classifier Training

Classification methods based on feature-level and sensitive cost functions are widely used to deal with problems related to unbalanced datasets [42]. In this context, the decision to implement cost-sensitive learning with a ratio of 5:1 in classifier training was based on the unbalanced nature of the dataset. The results shown in the previous section in Table 5, Table 6, Table 7, Table 8, Table 9 and Table 10 were derived from training of ML models on unbalanced data.

The cost ratio was experimentally deduced to find a balance between sensitivity and specificity and to avoid model overfitting. For the first participant, there were more migraine samples than non-migraine samples, while for all other participants it was the reverse, with fewer migraine samples. The goal of using cost-sensitive learning was to enhance the sensitivity of the predictive model to migraine samples, with the aim of improving the model’s precision in identifying migraine cases.

### 4.4. Comparative Analysis of Classifier Learning with Cost-Sensitive Approach

**Figure A1:**
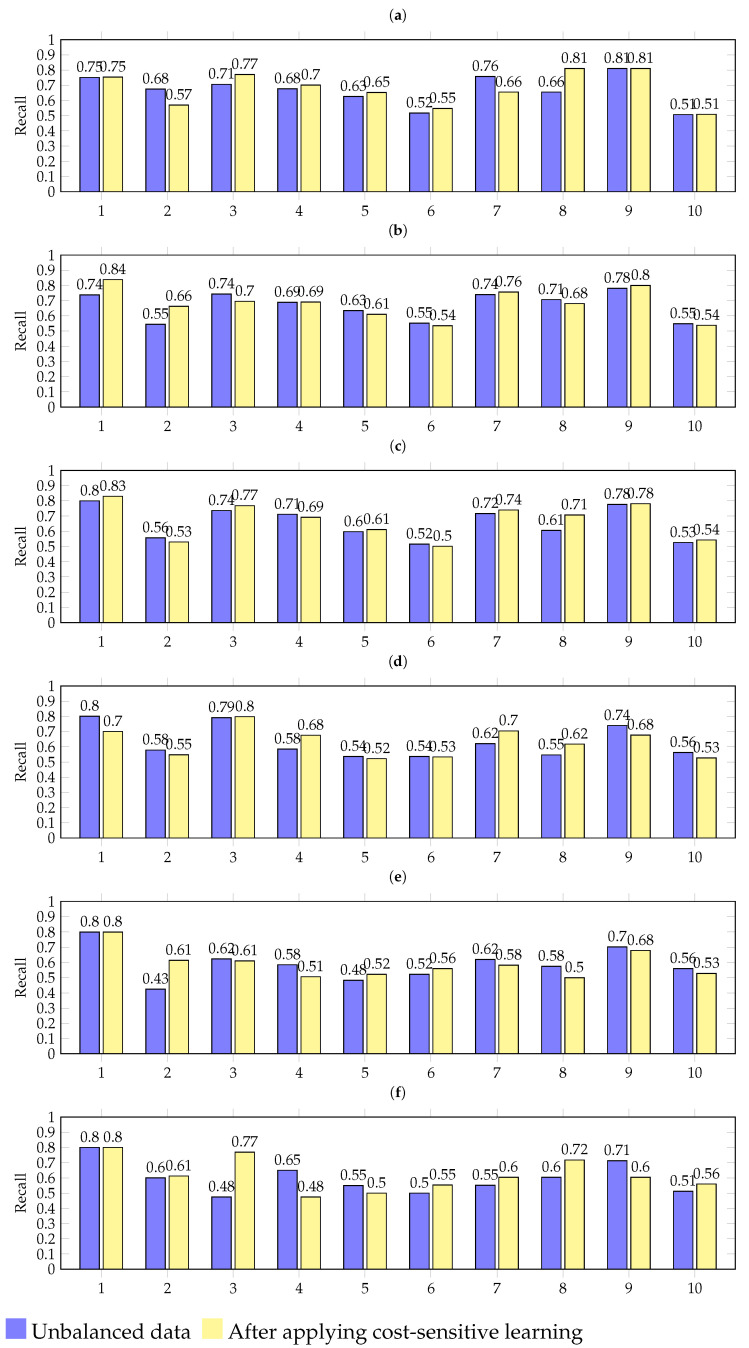
Comparison of recall for each participant before and after applying cost-sensitive learning: (**a**) 5 min analysis frame; (**b**) 10 min analysis frame; (**c**) 30 min analysis frame; (**d**) 60 min analysis frame; (**e**) 90 min analysis frame; (**f**) 120 min analysis frame.

Cost-sensitive learning was introduced in classifier training to achieve a ratio of 5:1. The results of the recall metrics of classifiers trained with cost-sensitive learning and unbalanced data are shown in Figure A1.

The application of cost-sensitive learning in classifier training generally led to an improvement in recall for participants who initially had lower recall metrics with unbalanced data. For example, participants 1, 3, and 10 showed increases in recall in various analysis frames, indicating that cost-sensitive learning effectively improved the model’s ability to identify migraine instances. However, some participants, such as 2 and 8, experienced a decrease in recall in most analysis frames, suggesting a potential trade-off when applying this approach. For others, such as participants 5 and 9, the recall metrics remained largely unchanged, indicating that the effect of cost-sensitive learning can be participant-specific. These variations in recall performance could be attributed to inherent differences in individual physiological responses and the complexity of their data patterns. In general, the cost-sensitive approach appears to benefit those with initially lower recall, highlighting its utility in improving migraine detection in unbalanced datasets.

### 4.5. ANOVA Feature Analysis: Comparison of Pre-Migraine Night with Night before a Migraine-Free Day

To evaluate the analysis of various features during the pre-migraine night and the night before a migraine-free day, an analysis of variance (ANOVA) was performed [43]. Based on the results of ANOVA, heat maps were generated showing the F-statistic and the *p*-value for each signal feature, as shown in Figure 3, Figure 4, Figure 5, Figure 6, Figure 7, Figure 8, Figure 9, Figure 10, Figure 11, Figure 12, Figure 13 and Figure 14.

The F-statistic measures the ratio of the variability between the group means to the variability within the groups. A higher F-statistic indicates that the group means are significantly different from each other relative to the variability within the groups. In this context, it helps determine whether there are significant differences in the feature values between nights marked with 0 and those with 1. The F-statistic in ANOVA is calculated as

(2) $$F=\frac{{\mathrm{MS}}_{\mathrm{between}}}{{\mathrm{MS}}_{\mathrm{within}}},$$

where

(3) $${\mathrm{MS}}_{\mathrm{between}}=\frac{{\mathrm{SS}}_{\mathrm{between}}}{{\mathrm{df}}_{\mathrm{between}}},$$

(4) $${\mathrm{MS}}_{\mathrm{within}}=\frac{{\mathrm{SS}}_{\mathrm{within}}}{{\mathrm{df}}_{\mathrm{within}}}.$$

${\mathrm{SS}}_{\mathrm{between}}$ (sum of squares between groups) is the variability due to the interaction between the groups. ${\mathrm{SS}}_{\mathrm{within}}$ (sum of squares within groups) is the variability within each group. ${\mathrm{df}}_{\mathrm{between}}$ and ${\mathrm{df}}_{\mathrm{within}}$ are the degrees of freedom between and within groups, respectively. These formulas were derived and used according to the principles outlined in [44,45].

The *p*-value indicates the probability that the observed differences between the group means occur by chance. A lower *p*-value suggests that the differences between the groups are statistically significant. In this study, it helps confirm whether the features vary significantly between the two types of nights.

The *p*-value is calculated on the basis of the F-distribution:

(5) p-value=P(F≥observedF).

This is the probability that the observed F-statistic would be as extreme as or more extreme than what was actually observed under the null hypothesis.

**Figure 3:**
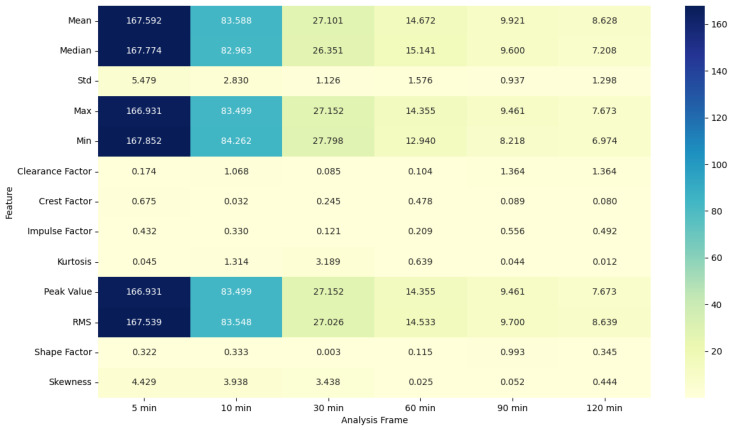
ANOVA F-statistic analysis of electrodermal activity features across various analysis frames.

**Figure 4:**
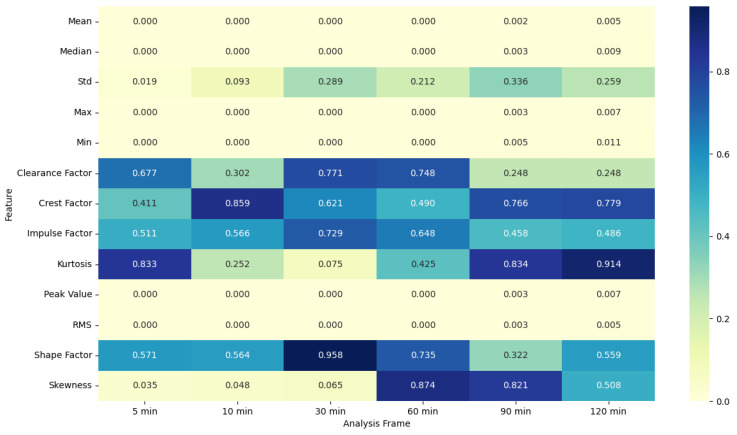
ANOVA *p*-value analysis of electrodermal activity features across various analysis frames.

The ANOVA F-statistic and *p*-value heat maps (Figure 3 and Figure 4) for the EDA features indicate that the shorter analysis frames of 5 and 10 min exhibit higher F-statistic values and significant *p*-values (less than 0.05) for several features such as mean, median, max, min, peak value, and RMS. This suggests that these features are significantly different when comparing pre-migraine nights to migraine-free nights within these shorter time frames. As the analysis frame length increases to 30 min, 60 min, 90 min, and 120 min, the F-statistic values decrease, and *p*-values rise above the significance threshold, indicating a reduced variance and less significant differences.

Similarly, for PR features, the F-statistic values are higher, and *p*-values are significant for shorter analysis frames of 5 and 10 min, particularly for features such as the clearance factor and the shape factor. This trend diminishes with longer analysis frames, where the F-statistic values drop and *p*-values become non-significant, highlighting that shorter frames are more effective for capturing significant variations in PR related to migraines.

**Figure 7:**
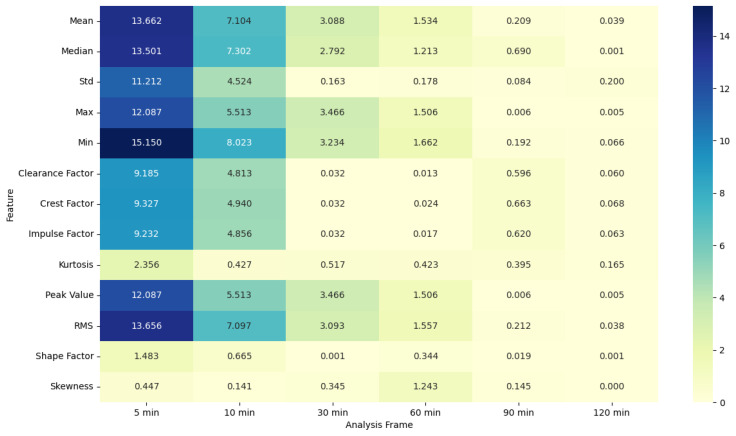
ANOVA F-statistic analysis of skin temperature features across various analysis frames.

**Figure 8:**
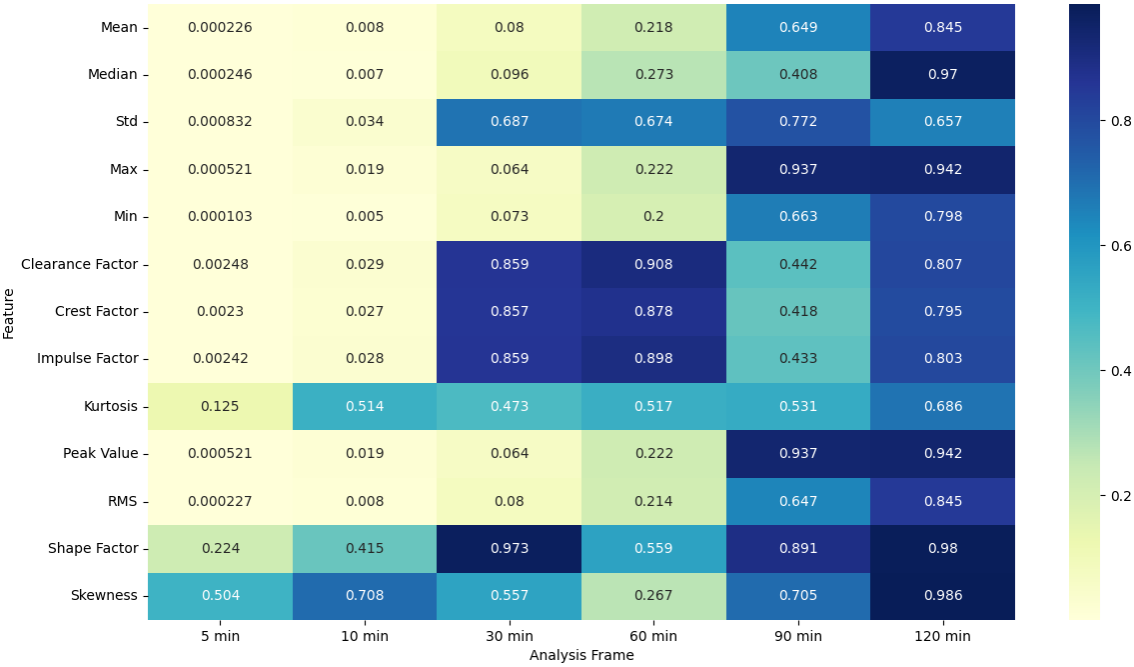
ANOVA *p*-value analysis of skin temperature features across various analysis frames.

For SkinTEMP features, the ANOVA results in Figure 7 and Figure 8 show significant F-statistic values and low *p*-values for shorter frames, indicating notable differences in features such as mean, median, Std, max, min, clearance factor, crest factor, impulse factor, peak value, and RMS during pre-migraine nights compared to migraine-free nights. As the frame lengthens, the significance of these differences reduces, as evidenced by lower F-statistic values and higher *p*-values.

MET features also display higher F-statistic values and significant *p*-values for shorter frames, with features such as mean, median, min, clearance factor, crest factor, impulse factor, and shape factor showing notable differences. Longer frames see a decline in both the F-statistic values and the significance of the *p*-values, suggesting that the predictive power of these features decreases over longer periods.

For ACT features, shorter analysis frames again show higher F-statistic values and significant *p*-values for features like mean, median, Std, max, min, peak value, and RMS. This trend aligns with previous observations, where longer analysis frames lead to a reduction in significance and variance, indicating that shorter frames are more suitable for capturing relevant differences.

**Figure 13:**
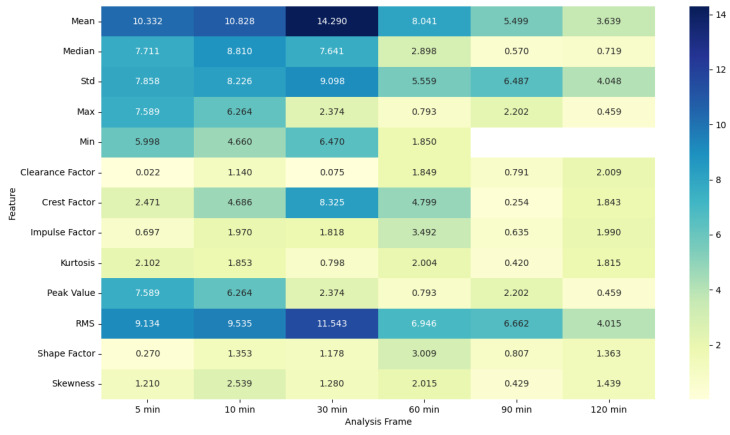
ANOVA F-statistic analysis of accelerometer features across various analysis frames.

**Figure 14:**
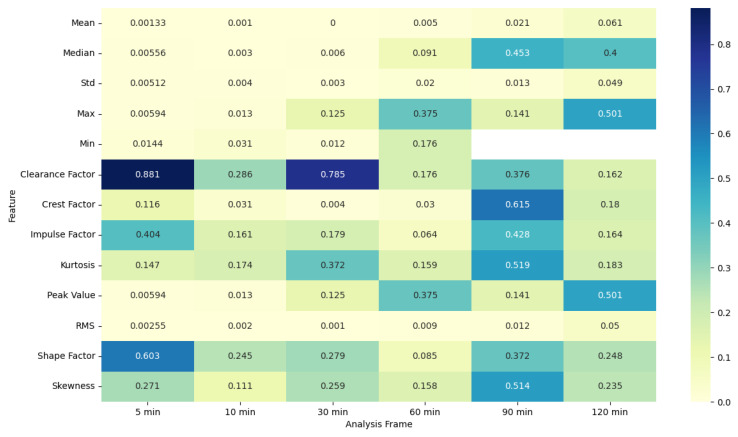
ANOVA *p*-value analysis of accelerometer features across various analysis frames.

The ANOVA results for the Acc features, as shown in Figure 13 and Figure 14, further reinforce the pattern, with higher F-statistic values and significant *p*-values for shorter frames of 5 and 10 min, particularly for features such as mean, median, Std, max, min, peak value, and RMS. As the analysis frames extend to 30 min, 60 min, 90 min, and 120 min, the statistical significance wanes, as reflected by lower F-statistic values and higher *p*-values.

The ANOVA analysis clearly indicates that shorter analysis frames of 5 and 10 min are more effective in capturing significant variations in physiological features between pre-migraine nights and migraine-free nights. These shorter frames exhibit higher F-statistic values and significant *p*-values for various characteristics, suggesting that they contain more relevant information for the prediction of migraines. As the analysis frame length increases, the significance of the differences diminishes, pointing towards a reduced predictive power over longer periods. This insight is important for developing effective migraine prediction models, emphasizing the importance of selecting appropriate analysis frame lengths to maximize predictive accuracy and clinical relevance.

EDA, SkinTEMP, and Acc features show the highest F-statistic values and the most significant *p*-values in the 5 and 10 min frames, making them particularly useful for early detection of migraines. MET and ACT features also show significant differences, especially in the 5 and 30 min frames, further indicating their potential utility in migraine prediction. These findings suggest that shorter analysis frames are generally more effective, and specific signals such as EDA, SkinTEMP, and Acc data are particularly valuable for accurate and timely migraine prediction.

### 4.6. Generalized Model for Predicting Migraines

The final stage of the study involved the training of ML models on data from the ten participants using cost-sensitive learning with a ratio of 5:1 to evaluate the significance of performance metrics. Among all the models evaluated, XGBoost demonstrated the best performance for the generalized model in terms of F1-score across all analysis frames, highlighting its robustness and effectiveness in predicting migraines regardless of the duration of the analysis frame, as shown in Table 11.

However, the clinical implications of these metrics, particularly recall, warrant careful consideration. Although XGBoost consistently provided the best F1-scores, the recall values indicate significant limitations in the model’s sensitivity, especially for longer analysis frames. Shorter analysis frames of 5 and 10 min offer relatively better performance, but still show a need for improvement in recall to ensure clinically effective migraine prediction.

These results suggest that the current generalized model, while robust in certain metrics, may not be sufficiently sensitive for clinical application, as it risks failing to detect a substantial number of true migraine events. Consequently, there is a pressing need to enhance the model’s sensitivity and ability to distinguish between migraine and non-migraine events. This could involve incorporating additional features, further optimizing the model, or integrating it with other predictive methods to improve its overall effectiveness in a clinical setting.

## 5. Discussion

The primary objective of this study was to explore the impact of the duration of the analysis frame and specific features on migraine prediction using wearable biosensor data. By investigating various physiological, activity-based, and signal processing metrics, we identified effective classifiers for different analysis intervals. Focusing on pre-migraine nights was crucial for understanding the patterns preceding migraine attacks while minimizing the confounding effects of physical activity on biomarkers.

Our results demonstrated that shorter analysis frames, specifically 5 and 10 min, yielded higher F1-scores and recall metrics among various participants. This finding suggests that these shorter frames are more effective in capturing the subtle physiological changes that precede migraine attacks. However, the study also highlighted the importance of balancing sensitivity and specificity, particularly in the context of migraine detection, where false positives and false negatives have distinct implications for patient care.

The use of cost-sensitive learning with a 5:1 ratio was a key strategy to address the unbalanced nature of the dataset. This approach improved recall metrics for several participants, enhancing the model’s ability to detect migraine instances. However, the effectiveness of this method varied among the participants, indicating the need for personalized approaches in the training of the classifier.

Feature extraction played an important role in this study and traditional statistical metrics such as minimum and median values proved to be more predictive than unconventional features. This emphasizes the importance of using a comprehensive range of features to build robust predictive models. Furthermore, the integration of frequently updated raw data during the prodromal phase was found to be beneficial in capturing the significant effects of daily activities on physiological parameters, thus improving the predictive power of the model.

Compared to the reviewed studies, our results align with those found by [18], which highlighted the importance of a multivariate analysis that involves the HR, EDA, SkinTEMP, and SpO2 signals for the prediction of migraine. They found that combining these signals improved prediction accuracy, although with a relatively high false positive rate. Our research further supports these findings by confirming that the EDA, SkinTEMP, and Acc data are significant predictors, especially when analyzed in shorter time frames.

Furthermore, the researchers in [25] demonstrated the effectiveness of personalized prediction models using hemodynamic signals, including TEMP, HR, and EDA, obtained from a wrist wearable device. They achieved a sensitivity of 95% in predicting migraine attacks with LSTM models. Our study supports the use of similar physiological signals and emphasizes the utility of shorter analysis frames to improve predictive accuracy. However, it is important to note that [25] trained their data with pain–no-pain balancing, which can lead to pseudo-high predictive accuracy. Thus, finding the best balancing approach and testing it on separate datasets is crucial. We consider investigating LSTM with other balancing methods.

The methodology of the study, including the exclusion of high-missing-value signals such as PRV and RR, and the application of normalization, ensured the reliability of the processed data. The decision to focus on nocturnal sleep data, segmented into various analysis frames, provided a robust framework for feature extraction and model training.

## 6. Research Limitations

Despite the promising results, several limitations must be acknowledged. First, the absence of a control group without migraines limits the generalizability of the findings. The study design relied on baseline data from nights before migraine-free days, which may not fully represent the variability in physiological signals among the general population.

Second, the sample size of ten participants, while sufficient for preliminary analysis, is relatively small to develop generalized predictive models. Future research should involve a larger and more diverse participant pool to enhance the robustness and applicability of the models.

Third, the study did not incorporate other potential migraine triggers, such as meteorological conditions, space weather, or menstrual cycles, which could influence the accuracy of the predictive models. Including these factors in future research could provide a more comprehensive understanding of migraine triggers and improve the accuracy of prediction.

Lastly, while the study focused on nocturnal sleep data, the possible impact of daytime activities and stress levels on physiological signals was not explored. Future studies should consider incorporating data from both daytime and nighttime periods to develop more holistic predictive models.

Furthermore, the study examined a limited set of ML algorithms, specifically XGBoost, HistGradientBoosting, Random Forest, SVM, and KNN. Research is constrained by this selection, and exploring a wider range of classifiers, including newer and more sophisticated algorithms, is necessary to fully understand the potential for accurate migraine prediction. Future studies should investigate other classifiers to provide a more comprehensive evaluation of different modeling approaches and their effectiveness in predicting migraines.

## 7. Conclusions

This study has demonstrated the potential of using wearable biosensor technology and ML models to predict migraine attacks by analyzing physiological signals during nocturnal sleep periods. The findings highlight the importance of shorter analysis frames (5 and 10 min) in capturing pre-migraine abnormalities, with features such as EDA, SkinTEMP, and Acc data showing the highest predictive power.

Although the use of cost-sensitive learning improved the recall metrics for several participants, the variability in effectiveness underscores the need for personalized approaches in classifier training. The study’s emphasis on traditional statistical metrics for feature extraction provides a solid foundation for future research in this area.

To enhance the clinical applicability of migraine prediction models, future studies should involve a larger and more diverse group of participants, incorporate additional migraine triggers, and explore the integration of physiological data during the day and at night. The development of adaptive self-learning models that account for individual variability and external factors holds promise for more personalized and effective migraine management strategies.

In conclusion, the integration of wearable technology and ML offers a promising pathway toward early detection and prevention of migraine attacks. Continued research and refinement of predictive models are essential to realize the full potential of this approach in clinical settings, ultimately improving the quality of life of individuals affected by migraines and other neurological conditions.

Future work should focus on expanding the pool of participants to improve the generalizability of predictive models and incorporate a wider range of migraine triggers, such as meteorological conditions and menstrual cycles. Furthermore, future studies should explore both daytime and nighttime physiological data, as well as a wider array of machine learning algorithms, to develop more robust and accurate migraine prediction models.

## Figures and Tables

**Figure 5:**
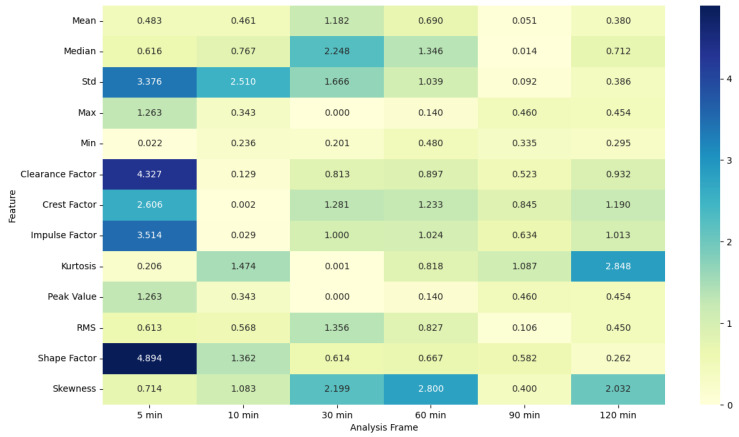
ANOVA F-statistic analysis of pulse rate features across various analysis frames.

**Figure 6:**
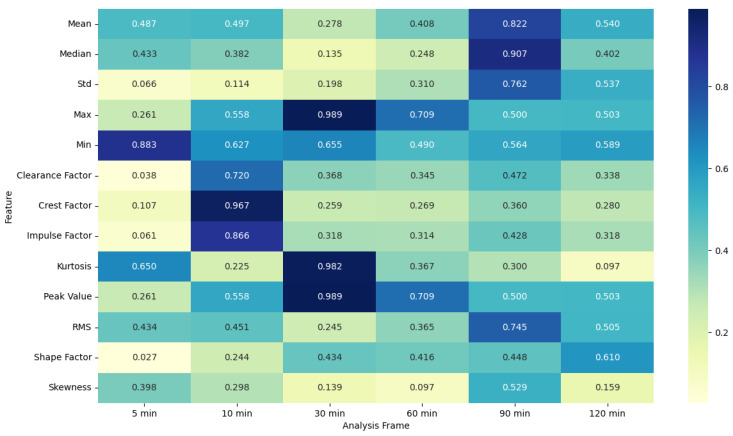
ANOVA *p*-value analysis of pulse rate features across various analysis frames.

**Figure 9:**
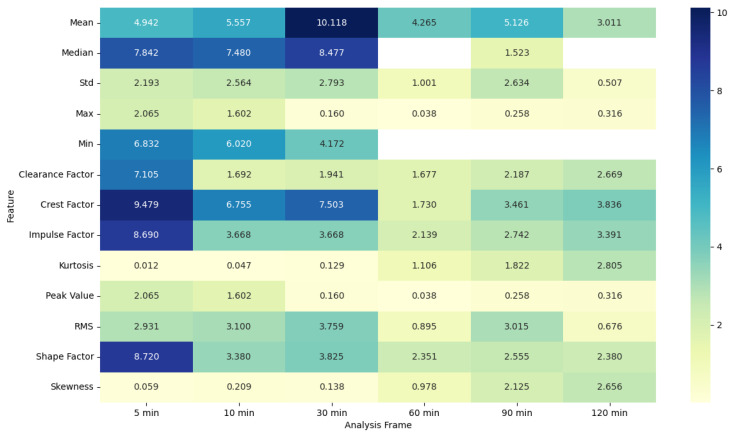
ANOVA F-statistic analysis of metabolic equivalent features across various analysis frames.

**Figure 10:**
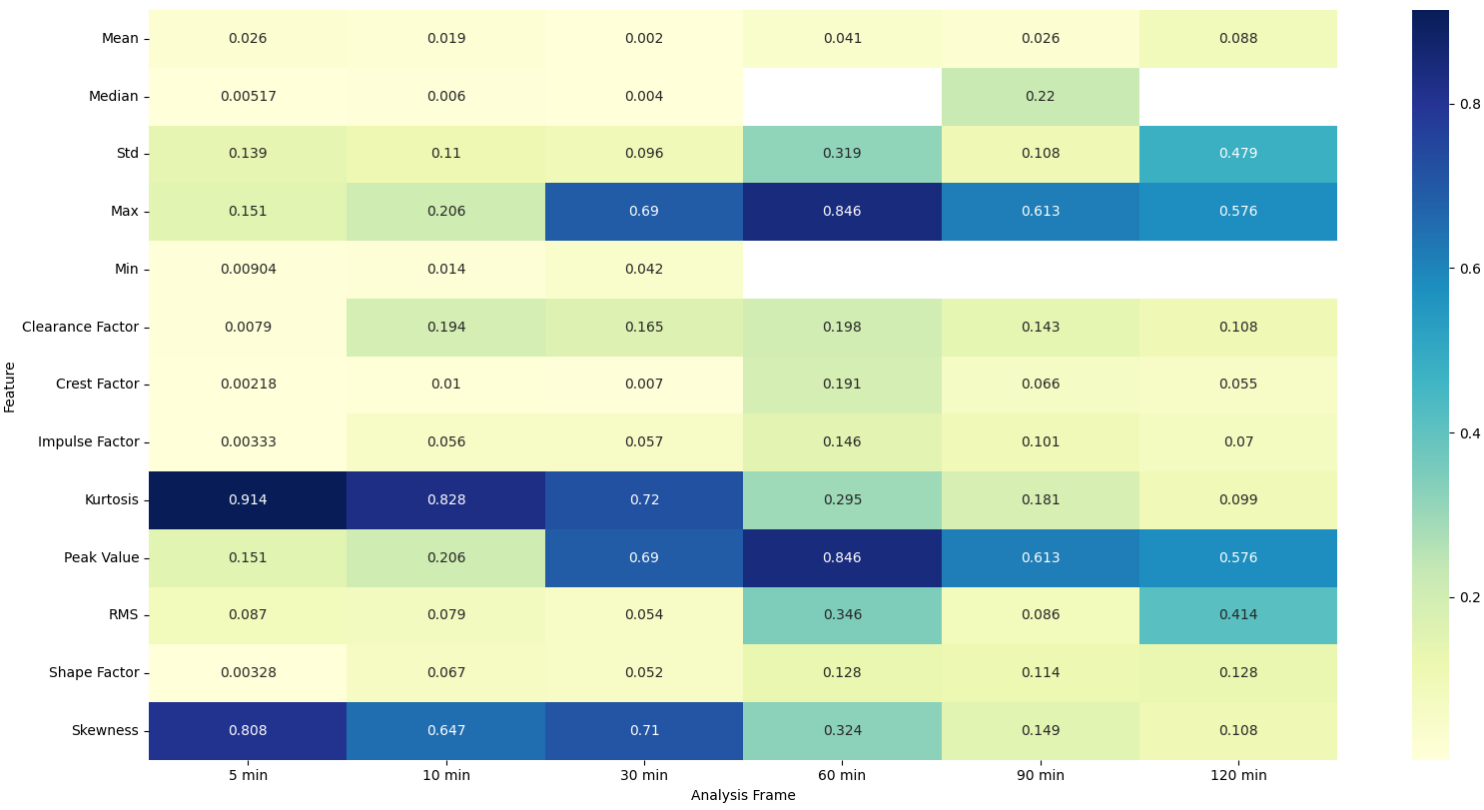
ANOVA *p*-value analysis of metabolic equivalent features across various analysis frames.

**Figure 11:**
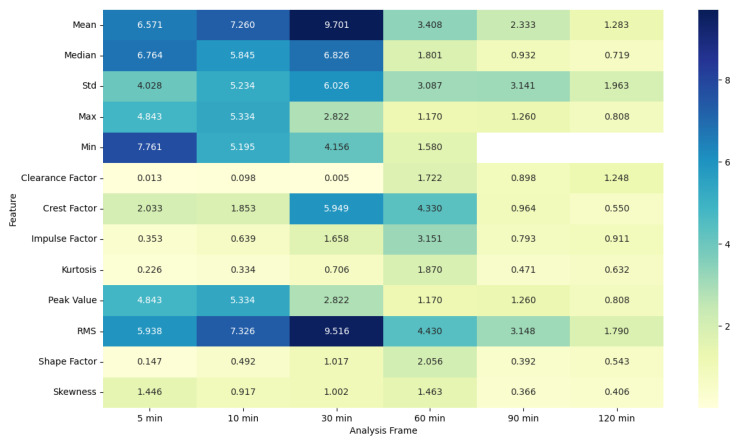
ANOVA F-statistic analysis of activity counts features across various analysis frames.

**Figure 12:**
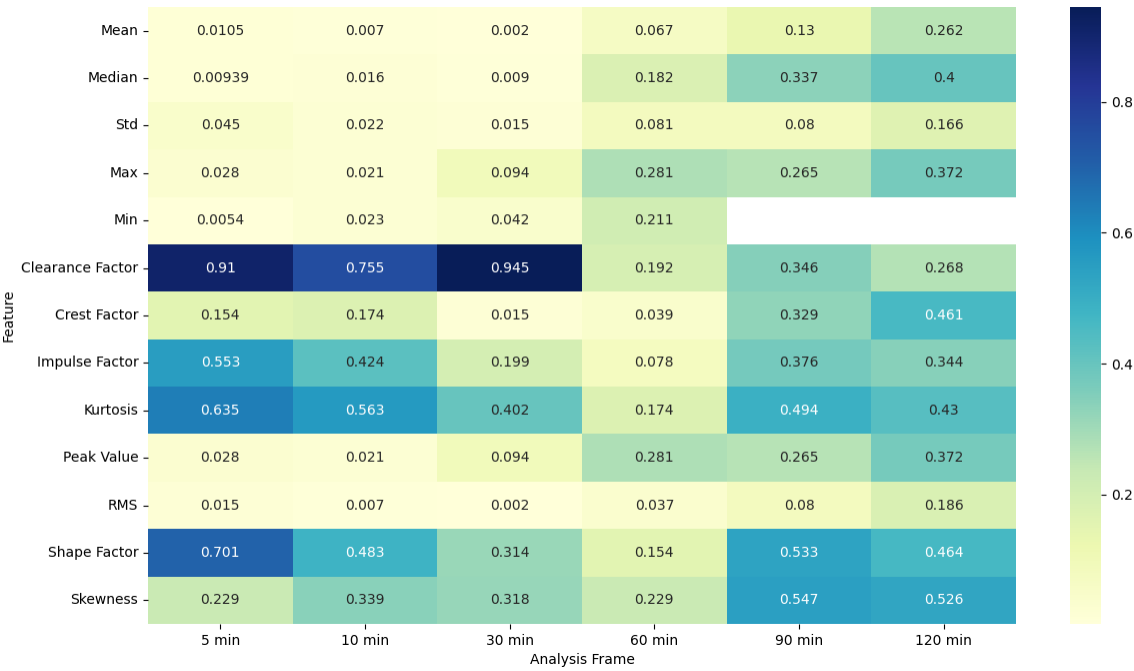
ANOVA *p*-value analysis of activity counts features across various analysis frames.

**Table 1:** Demographic information and monitoring days of study participants.

Participant	Age	Sex	Migraine Type	Migraine Duration (Years)	Monthly Migraine Headache Days	Monitoring Days
1	30	M	Without aura	5	7	7
2	29	M	With and without aura	8	6	21
3	33	F	Without aura	23	14	52
4	29	M	Without aura	23	5	33
5	22	F	Without aura	10	8	33
6	31	F	Without aura	10	12	68
7	39	F	Without aura	5	6	20
8	29	F	Without aura	20	5	40
9	62	M	Without aura	29	7	25
10	26	M	Without aura	9	6	23

Note: F—female, M—male.

**Table 2:** Percentage of missing values.

Signal	% of Missing Values
Accelerometers	14.23%
Activity count	14.23%
Electrodermal activity	14.23%
Metabolic equivalent of task	14.23%
Pulse rate variability	75.20%
Pulse rate	14.23%
Respiratory rate	70.81%
Sleep detection stage	14.23%
Step count	14.23%
Skin temperature	14.23%
Wearing detection	13.06%

Note: The yellow highlight indicates significantly high missing values.

**Table 3:** Feature ranking and recurrence of signal source.

Signal Source	Importance Score
EDA	0.509
Pulse rate	0.735
Accelerometers	0.333
Activity count	0.295
MET	0.376
Skin temperature	0.803

**Table 4:** Feature ranking and importance scores for statistical and signal processing measurements.

Feature Name	Importance Score
Mean	0.535
Median	0.576
Standard deviation	0.410
Max	0.493
Min	0.743
Clearance factor	0.438
Crest factor	0.479
Impulse factor	0.458
Kurtosis	0.319
Peak value	0.493
RMS	0.507
Shape factor	0.493
Skewness	0.306

**Table 5:** Best classifiers for each participant based on F1-score for 5 min analysis frame.

Participant	Model	Accuracy	Precision	Recall	F1-Score
1	Random Forest	0.853	0.852	0.751	0.757
2	SVM	0.675	0.677	0.675	0.671
3	Random Forest	0.860	0.849	0.706	0.731
4	XGBoost	0.868	0.753	0.677	0.689
5	Random Forest	0.827	0.757	0.625	0.642
6	XGBoost	0.868	0.540	0.517	0.510
7	XGBoost	0.878	0.608	0.507	0.494
8	HistGradientBoosting	0.829	0.785	0.758	0.768
9	HistGradientBoosting	0.863	0.819	0.655	0.698
10	Random Forest	0.834	0.846	0.810	0.829

**Table 6:** Best classifiers for each participant based on F1-score for 10 min analysis frame.

Participant	Model	Accuracy	Precision	Recall	F1-Score
1	XGBoost	0.893	0.876	0.838	0.848
2	KNN	0.664	0.698	0.663	0.654
3	Random Forest	0.846	0.803	0.696	0.725
4	XGBoost	0.853	0.738	0.691	0.709
5	XGBoost	0.794	0.665	0.610	0.619
6	XGBoost	0.883	0.605	0.535	0.537
7	HistGradientBoosting	0.884	0.582	0.525	0.522
8	HistGradientBoosting	0.847	0.809	0.756	0.776
9	HistGradientBoosting	0.868	0.821	0.681	0.717
10	Random Forest	0.820	0.798	0.800	0.802

**Table 7:** Best classifiers for each participant based on F1-score for 30 min analysis frame.

Participant	Model	Accuracy	Precision	Recall	F1-Score
1	Random Forest	0.900	0.943	0.800	0.802
2	XGBoost	0.589	0.590	0.557	0.555
3	HistGradientBoosting	0.862	0.780	0.736	0.738
4	HistGradientBoosting	0.888	0.855	0.711	0.753
5	XGBoost	0.784	0.642	0.597	0.608
6	XGBoost	0.891	0.684	0.516	0.508
7	Random Forest	0.889	0.845	0.507	0.484
8	HistGradientBoosting	0.821	0.781	0.716	0.729
9	HistGradientBoosting	0.840	0.749	0.606	0.628
10	Random Forest	0.795	0.789	0.776	0.775

**Table 8:** Best classifiers for each participant based on F1-score for 60 min analysis frame.

Participant	Model	Accuracy	Precision	Recall	F1-Score
1	Random Forest	0.817	0.942	0.800	0.766
2	XGBoost	0.639	0.613	0.578	0.532
3	HistGradientBoosting	0.892	0.669	0.563	0.574
4	XGBoost	0.848	0.782	0.585	0.597
5	Random Forest	0.795	0.605	0.544	0.536
6	XGBoost	0.887	0.651	0.535	0.532
7	Random Forest	0.886	0.943	0.500	0.484
8	KNN	0.750	0.738	0.620	0.625
9	Random Forest	0.829	0.753	0.546	0.580
10	Random Forest	0.752	0.778	0.738	0.767

**Table 9:** Best classifiers for each participant based on F1-score for 90 min analysis frame.

Participant	Model	Accuracy	Precision	Recall	F1-Score
1	XGBoost	0.833	0.917	0.800	0.747
2	Random Forest	0.533	0.520	0.425	0.429
3	HistGradientBoosting	0.894	0.688	0.560	0.574
4	XGBoost	0.848	0.782	0.585	0.597
5	Random Forest	0.740	0.647	0.482	0.505
6	KNN	0.885	0.785	0.522	0.506
7	Random Forest	0.886	0.943	0.500	0.470
8	KNN	0.750	0.738	0.620	0.625
9	Random Forest	0.838	0.668	0.575	0.522
10	XGBoost	0.716	0.717	0.702	0.696

**Table 10:** Best classifiers for each participant based on F1-score for 120 min analysis frame.

Participant	Model	Accuracy	Precision	Recall	F1-Score
1	XGBoost	0.800	0.900	0.800	0.733
2	KNN	0.680	0.675	0.600	0.613
3	KNN	0.890	0.705	0.553	0.560
4	RandomForest	0.858	0.945	0.650	0.632
5	RandomForest	0.757	0.689	0.550	0.474
6	RandomForest	0.880	0.945	0.500	0.471
7	KNN	0.856	0.678	0.512	0.494
8	XGBoost	0.727	0.586	0.551	0.546
9	XGBoost	0.820	0.695	0.604	0.595
10	HistGradientBoosting	0.724	0.754	0.713	0.699

**Table 11:** Performance metrics of the generalized migraine prediction model using XGBoost for different analysis frame durations.

Analysis Frame	Accuracy	Precision	Recall	F1-Score
5 min	0.806	0.638	0.595	0.607
10 min	0.811	0.640	0.587	0.599
30 min	0.816	0.634	0.563	0.572
60 min	0.803	0.585	0.535	0.533
90 min	0.790	0.517	0.508	0.495
120 min	0.794	0.553	0.511	0.498

## Data Availability

The datasets presented in this article are not available, the data are part of an ongoing study.

## Abbreviations

The following abbreviations are used in this manuscript:

Acc	Accelerometer
ACT	Activity count
AI	Artificial intelligence
ANS	Autonomic nervous system
bpm	Bats per minute
ECG	Electrocardiogram
EDA	Electrodermal activity
HR	Heart rate
MET	Metabolic equivalent of task
ML	Machine learning
NaN	Not a number
PPG	Photoplethysmography
PR	Pulse rate
PRV	Pulse rate variability
RMSSD	Root mean square of successive differences
RR	Respiratory rate
SC	Step count
SDS	Sleep detection stage
SkinTEMP	Skin temperature
SpO2	Peripheral capillary oxygen saturation
TEMP	Temperature

## Appendix A. Extended Data Visualization

This appendix provides extended data visualizations relating to the analysis presented in the main body of the paper. These visualizations offer a more in-depth view of the results.
